# Electropolymerization of Metallo-Octaethylporphyrins: A Study to Explore Their Sensing Capabilities

**DOI:** 10.3390/ma15196598

**Published:** 2022-09-23

**Authors:** Clésia C. Nascentes, Ivette Aguilar, Guzmán Gil-Ramírez, Jose Gonzalez-Rodriguez

**Affiliations:** 1 Department of Chemistry, Federal University of Minas Gerais, P.O. Box 702, Belo Horizonte 31270-901, Brazil; 2 School of Chemistry, University of Lincoln, Brayford Pool, Lincoln LN6 7TS, UK

**Keywords:** electropolymerization, metallo-octaethylporphyrins, UV-Vis, Raman, phenobarbital

## Abstract

The electropolymerization of metallo-octaethylporphyrins (OEP) containing copper, zinc or nickel metal were performed using cyclic voltammetry at three different potential ranges. The electropolymerized porphyrins were characterized by UV-Vis and Raman spectroscopies and the Soret band (393–445 nm) and Raman bands were used to assess the degree of electropolymerization obtained. The application for an analytical use of the modified electrodes to determine phenobarbital in aqueous solution was evaluated. The electropolymerized CuOEP produced at potentials ranging from 0.0 to 2.2 V was the best performer with a limit of detection (LoD) of 10 mg L^−1^ (43.07 µM), a linear range of 10–150 mg L^−1^ (43.07 to 646 µM), an average precision of 4.3% (%RSD) and an average % recovery of 101.34%. These results indicate that the CuOEP-modified electrode is suitable for the analysis of phenobarbital in human samples, as the concentration range varies from 10 to 40 mg L^−1^ (43.07 to 172.27 µM), typically found in antiepileptic treatments, to those at the toxic level (172–258 µM) or lethal levels (345–650 µM).

## 1. Introduction

Porphyrins are a group of heterocyclic macrocycle organic compounds, composed of four modified pyrrole subunits interconnected at their carbon atoms via methine (=CH–) bridges [1]. Polymers of porphyrins and metalloporphyrins have been applied in different areas such as electronic devices, solar energy cells, catalysis and electrochemical sensors [2,3,4]. Different strategies allowing for the formation of porphyrins polymers in solution have been reported, however, in the electroanalysis field exists a particular interest in compounds that can be electropolymerized in order to obtain surface-modified electrodes [5,6,7]. In 1983, Macor and Spiro [8] reported for the first time electropolymerized porphyrins by the coupling of electrogenerated radical vinyl substituents.

The electrochemical strategy presents some advantages, such as: (i) it is an easy way to functionalize conductive surfaces with great precision; (ii) it allows for the formation of films with a good reproducibility and a controlled thickness; and (iii) it provides densely packed layers that facilitate the electron hopping process between macrocycles [9,10,11].

Electrochemical sensors based on porphyrins have been previously applied to the determination of inorganic and organic analytes. Chen and coworkers [6] prepared a selective sensor for the detection of explosives based on the electropolymerization of [meso-tetrakis (2-thienyl) porphyrin]. Another example of this use was reported by van Staden et al., where five different porphyrins were evaluated to prepare a sensor for the determination of ascorbic acid in pharmaceuticals, beverages and biological fluids [12].

The addition of a metal to the porphyrin structure has important implications, both structurally and from an electronic point of view. In this sense, the electropolymerization of some metalloporphyrins and the study of their structural characteristics has been a very active topic, and the literature shows examples of electrochemical methods where the understanding and structural characteristics achieved from the electropolymerization of porphyrins can vary depending on the experimental conditions used [13,14]. The concentrations and type of porphyrins and ligands, solvent, potential range, scan rate and electrode used as a support are some important variables to take into account.

The aim of this study is to obtain thin films of metal-containing (Ni, Cu and Zn) octaethylporphyrins (OEP) and bipyridine by electropolymerization through anodic oxidation performed on an indium tin oxide (ITO) electrode substrate. The electrochemical properties of these polymer films is then reported for the corresponding copper and zinc complexes at three different potential ranges. To the best of our knowledge, this is the first time nickel octaethylporphyrin has been electropolymerized on this surface, adding to the list of metalloporphyrins capable of this reactivity. The polymer films were characterized by UV-Vis spectroscopy. Raman spectroscopy was performed for the first time to evaluate the performance of the electropolymerization under these conditions, adding to the vibrational behavior of the different electropolymers generated. Finally, for the first time the application of the porphyrin-modified ITO electrodes, used as an analytical tool to determine phenobarbital in aqueous solution, is evaluated.

## 2. Materials and Methods

**Materials** All solvents and reagents were analytical grade and used without further purification. The phenobarbital sodium salt, 4,4′-bipyridyl, 1,2-dichloroethane, dichloromethane (DCM), tetrabutylammonium hexafluorophosphate, nickel acetylacetonate, copper acetate, zinc acetate, sodium chloride (NaCl), glacial acetic acid, potassium chloride (KCl), sodium perchlorate (NaClO_4_), lithium perchlorate (LiClO_4_), sodium acetate and boric acid were obtained from Sigma-Aldrich (UK). The octaethylporphyrin (OEP) was purchased from Porphychem (Dijon, France). The phosphoric acid, hydrochloric acid and potassium hydroxide were all purchased from Fisher Scientific (UK). A Britton–Robinson buffer solution was prepared using phosphoric acid, glacial acetic acid and sodium chloride; the pH value was adjusted with NaOH and HCl. The phenobarbital working solutions were freshly prepared daily.

**Porphyrin synthesis** The synthesis of the different porphyrins used, M-OEP (M = Cu(II), Ni(II) and Zn(II)), followed the protocols established by Gil-Ramirez et al. [15] for zinc, and Davis et al. for copper and nickel [16].

**Electropolymerization** The electrochemical experiments were performed using an Autolab (Ecochemie model Pgstat3) instrument attached to a computer with the proper software (NOVA) for the total control of the experiments and data acquisition. A conventional three electrode system consisting of using one-side indium tin oxide coated (ITO Sigma-Aldrich surface resistivity 8–12 Ω/sq with a surface of 3 cm^2^) as a working electrode, an Ag/AgCl electrode as a reference electrode and a platinum electrode as an auxiliary electrode, were used for all the experiments. The electrochemical process was carried out under a nitrogen atmosphere in 1,2-C_2_H_4_Cl_2_ containing 0.1 M of tetraethylammonium hexafluorophosphate and 0.25 mM of M-OEP (M = H_2_, Cu(II), Ni(II), Zn(II)) and 4 mM of 4,4′-bipyridine (bpy). The electropolymerization was performed by cyclic voltammetry in three different potential ranges: −1.0 V to 1.7 V, 0 V to 1.6 V and 0 V to 2.2 V vs. Ag/AgCl at a scan rate of 0.1 V · s^−1^ for 25 cycles. After the electropolymerization, the working electrode was washed with deionized water to remove traces of the conducting salt present on the deposited film.

**Characterization** The polymers obtained were characterized by UV-Vis and Raman, Respectively. The UV-Vis analyses were performed on an Analytik Jena SP150 spectrometer (Thuringia, Germany). Glass plates covered with indium–tin–oxide (ITO) were used as blank (reference) and the modified electrodes were analyzed directly from 350 nm to 700 nm to obtain the UV–vis spectra of the electrochemically deposited polymers.

For comparison, a piece of the ITO was placed in contact with the solution used for electropolymerization for 5 min and after that time was removed and air dried. After drying, it was washed with deionized water and analyzed by UV-Vis. A solution of each of the porphyrin monomers in DCM was also analyzed. Raman analyses were performed in a LabRaman HR800 Raman setup (Horiba Jobin Ybon) equipped with a BX41 microscope (Olympus) using a 50x objective and a laser Quantum ltd ventus 532.04 nm. The following acquisition settings were used: an RTD (residence time distribution) exposure time of 10 s, accumulation number of 2 s and the exposure time of 10 s in order to improve the spatial resolution.

### Analytical Performance 

To demonstrate their potential analytical use and the effect of the different electropolymerization conditions, the different porphyrin-modified ITO electrodes produced were evaluated on their analytical performance for the determination of phenobarbital in aqueous solution. Phenobarbital solutions were prepared using LiClO_4_ 0.05 mol L^−1^ as the supporting electrolyte. The analytical signal was obtained using an Autolab (Ecochemie model Pgstat3) instrument with a conventional three electrode system consisting of a modified electrode as the working electrode, an Ag/AgCl electrode as the reference electrode and platinum as an auxiliary electrode. A CV was performed in the potential range from 0 V to 1.0 V with a maximum peak current at 0.564 V.

The modified electrode that presented a higher response was used to determine the main analytical characteristics of phenobarbital. In this study, we evaluated the linear range (0 to 150 mg L^−1^), limits of detection and quantification (from analysis of 10 blank solutions), precision and accuracy to phenobarbital in three levels (30.0; 60.0; and 118.0 mg L^−1^).

## 3. Results and Discussion

### 3.1. Electropolymerization 

**Figure 1:**
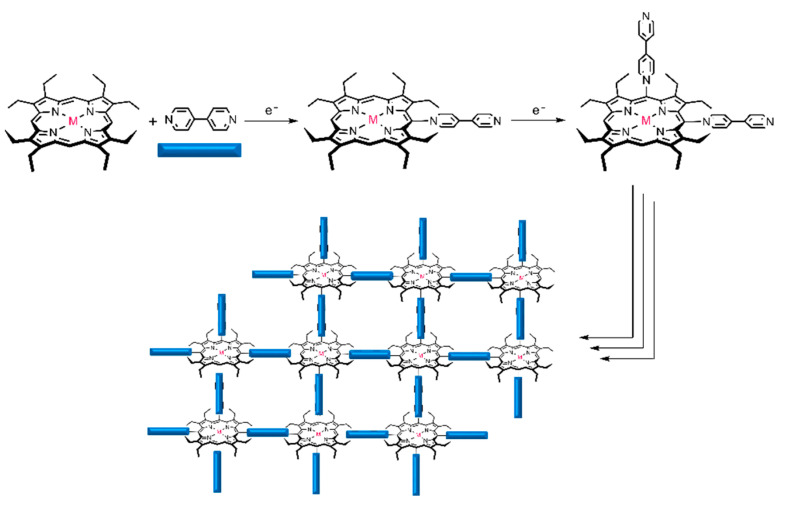
Representation of an alternating porphyrin–viologen copolymer creating a 2D layer.

The electropolymerization behavior of metalloporphyrins and bipyridine has been widely studied by Giradeau and Rhulmann [11,13,14]. In the case of the nucleophilic substitution of bipyridine with Zinc octaethyl porphyrin obtained by electrolysis, the potential applied correlates with the level of meso-substitution was achieved. The use of cyclic scanning voltammetry (0.1 V/s) on the ITO electrodes leads to the formation of an alternating porphyrin–viologen copolymer (Figure 1), as previously reported by Giradeau and Rhulmann [13].

The simple method used of coating electrodes with porphyrin polymers was proposed by Giraudeau and coworkers [17] and it is based on the electropolymerization of unmodified porphyrins, i.e., without the preliminary attachment of specific substituents on the porphyrin ring, using bipyridine as a spacer. Previously published reports indicated the electropolymerization mechanism as a nucleophilic substitution on porphyrins where the dication formation is important [17,18]. Indeed, 4,4′-bipyridine presents two accessible nucleophilic sites which can react one after the other with two porphyrin rings, and in this mechanism the electrogeneration of the radical cation of the porphyrin has been efficient (process ECEC) [19]. Other studies have showed that the potential ranges applied (positive, negative or both) in the electropolymerization process can affect the cyclic voltammograms profile and the polymer characteristics [20]. In this way, we performed a study to evaluate the effect of the potential range used for electropolymerization of octaethylporphyrins (OEP, CuOEP, NiOEP and ZnOEP) on the characteristics and analytical performance of the modified electrodes.

**Figure 2:**
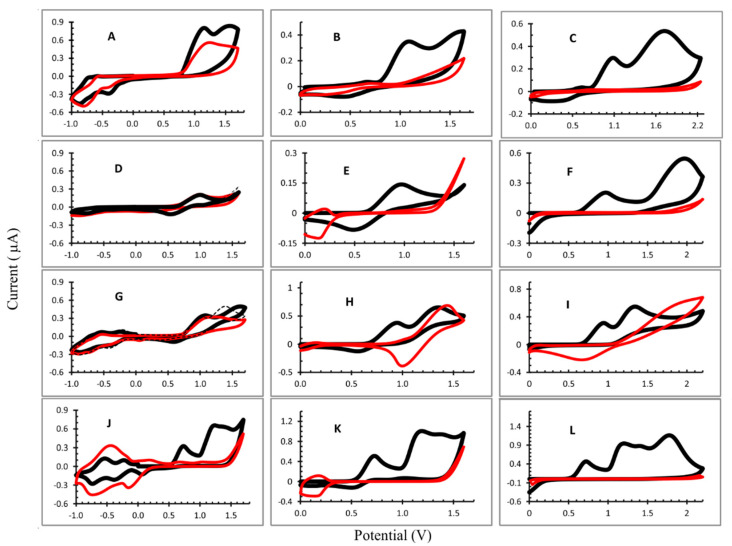
Cyclic voltammograms recorded during electropolymerization: (**A**) OEP^1^; (**B**) OEP^2^; (**C**) OEP^3^; (**D**) CuOEP^1^; (**E**) CuOEP^2^; (**F**) CuOEP^3^; (**G**) NiOEP^1^; (**H**) Ni OEP^2^; (**I**) Ni OEP^3^; (**J**) ZnOEP^1^; (**K**) ZnOEP^2^; and (**L**) Zn OEP^3^. Potential range: ^1^: −1.0 V to 1.7 V; ^2^: 0.0 V to 1.6 V; and ^3^: 0.0 V to 2.2 V. 1,2-C_2_H_4_Cl_2_ with 0.1 mol L^−1^ TBAP and 4 mmol L^−1^ bpy. Working electrode: ITO; scan rate: 0.1 V s^−1^. Black line: first cycle and red line: 25th cycle.

Three potential ranges were evaluated: −1 V to 1.6 V; 0 to 1.6 V; and 0 V to 2.2 V. The cyclic voltammograms obtained are shown in Figure 2 and the half-wave potentials in Table 1. The potential values of oxidation were measured on the first scan, because of the further polymerization of the porphyrins with the increased number of scans. 

In general, the CV profiles were different for each experiment performed. For the OEP electropolymerization (Figure 2A–C), the first oxidation peaks of macrocycle (radical cation) appear in the all potential ranges evaluated and the second peak (dication) can be observed when the potential ranged from 0 to 2.2 V (considering the first cycle). The reduction peaks were not observed due to the porphyrin, the radical anion and the dianion occuring in E < −1.0 V. The iterative scans lead to a gradual decrease in the oxidation peaks and an increase in the reduction peak is observed in −0.813 V (Figure 2A), attributed to the electron transfers centered on the electrogenerated viologen units being introduced between two porphyrins [19,21], evidencing the formation of the film on the ITO. The minimum current was obtained in the electrode produced in the potential range from 0 to 2.2 V (Figure 2C), indicating a better coverage of the ITO surface by the polymer film. 

In the formation of the poly-CuOEP, (Figure 2E), iterative scans between 0.0 V and +1.60 V led to a gradual decrease in the large oxidation and reduction peaks with E_½_ = 0.972 V and 0.486 V, respectively, that corresponded to the first reversible oxidation of macrocycle. Moreover, an increase in the peak at +0.137 V was observed, which most likely corresponded to the reduction of the pyridinium groups of the spacers (Py+/Py) and was assigned to the formation of the polymer film [19]. However, when the potential range was changed to −1.0 to 1.7 V, a different behavior was observed. The oxidation and reduction peaks of the radical cation and radical anion appeared at E_½_ = 1.043 V and 0.515 V, respectively. However, iterative scans did not lead to significative changes in the CV profile and a thin polymer film was expected to be formed in this condition (Figure 2D). In the potential range 0 V to 2.2 V (Figure 2F), the CV for the CuOEP presented two oxidation peaks (0.972 V and 1.978 V) in the first cycle. However, the first reduction peak did not appear and on the second cycle only could the dication peak could be seen; its signal decreased gradually and disappeared at the end of the electropolymerization process. On the ITO, a yellow polymer film can be observed under these experimental conditions.

The electropolymerization of the NiOEP at different potential ranges resulted in very distinct polymers. In fact, the site of electrooxidation in nickel(II) porphyrins and related macrocycles has been a point of discussion in the literature. Ni(II) porphyrins can present planar and nonplanar forms and the metal oxidation (Ni(II)/Ni(III)) in the electrochemical process has been described in the literature [16,21]. When the CV was obtained at the potential range from −1.0 V to 1.7 V, radical cation, dication and radical anion were observed in the first cycle (Figure 2G). From the second cycle, the peak of the cation radical did not change and the peak of the dication gradually decreased. After 25 cycles, the intensity of the radical cation peak was very similar to the first cycle and a slight difference was observed with the ITO surface. Moreover, the reduction peaks (−0.427 V and −0.872 V), attributed to the electron transfers centered on the electrogenerated viologen units introduced between two porphyrins, increased during the first ten cycles and thereafter decreased so that, after 25 cycles, there was not much difference in the current intensity at these two potentials. 

In the other two conditions used (only in positive potentials), the NiOEP film that formed had a dark color (Supplementary Information, Figure S1) and had a poor adherence to the surface of the ITO. The CV (Figure 2H,I) shows two oxidation peaks in the first cycle and, after the second cycle, the peak shifted to higher potentials which may be due to the oxidation of the metal (Ni(II)/Ni(III)), which is favored in the presence of bipyridine [22] forming [Ni(III)OEP]^+^. Besides that, a reduction peak appeared and increased after successive cycles (E_½_ = 1.01 V and E_½_ = 0.758 V, to potential ranges from 0.0 V to 1.6 V and 0.0 V to 2.2 V, respectively). This peak can be attributed to a reduction in the [Ni(III)OEP]+, that form the polymeric film, resulting in a dark color. 

The potential separation between the two ring-centered oxidations of many nonplanar nickel(II) porphyrins was often equal to zero in dichloromethane (CH_2_Cl_2_) containing 0.1 M of tetrabutylammonium perchlorate as the supporting electrolyte; i.e., the two one-electron oxidations were overlapped to give an overall two-electron transfer process in a single step [23]. In this way, from the second cycle we could only observe the oxidation peak of dication ([Ni(II)OEP]^2+^) that decreased with the iterative scans, and the reduction peak attributed to the radical anion formation [Ni(II)OEP]^+^ increased.

In the three conditions studied for the ZnOEP electropolymerization, the radical cation and dication oxidation peaks could be observed (Figure 2J–L and Table 1). In addition, when the applied potential was 0 to 2.2 V (Figure 2L), a third oxidation peak was observed at 1.775 V vs. Ag/AgCl. Reduction peaks are not observed in this case, demonstrating the irreversibility of the reaction. 

In the Figure 2J (−1.0 V to 1.7 V), three one-electron reduction peaks were observed at ca. 0.061 V, −0.388 V and −0.737 V vs. Ag/AgCl which corresponded, respectively, to the typical electronic transfers onto the viologen linkers and bipyridinium substituents [19,24], as already discussed. For all of the conditions studied, the ZnOEP electropolymerization resulted in a decrease in the oxidation peaks corresponding to the radical cation and dication, proving the formation of polymers, which can also be visually evaluated by the reddish coloration observed on the surface of the ITO electrode.

### 3.2. UV-Vis and Raman Characterization

**Figure 3:**
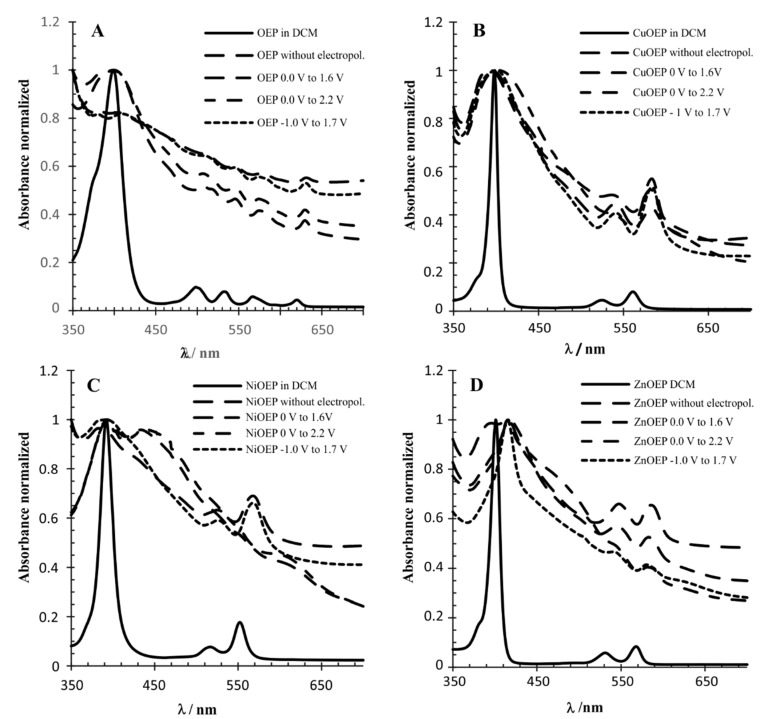
UV-vis normalized absorption spectra of metalloporphyrins monomers and polymers: (**A**) OEP; (**B**) CuOEP; (**C**) NiOEP; and (**D**) ZnOEP.

The polymers were characterized by UV-visible absorption spectroscopy (Table 2). The spectra were recorded on a polymer-coated ITO electrode and then compared to the spectra of the monomers deposited on the ITO and monomers in the solution (Figure 3). The intensity of all spectra was normalized. The superimposition showed that the half-line width of the Soret band, attributed to the main porphyrin-based π-π* electronic transition, is larger in metalloporphyrins (monomers) deposited onto the ITO than these compounds dissolved in DCM. This effect has been attributed to the electropolymerization process when comparing just the polymers on the ITO with the monomers in the solution [11,17]. However, this experiment demonstrated that the preponderant effect in this case was the solvation that interferes in the intra- and inter-molecular exciton coupling between the porphyrin molecules. The UV-Vis absorption spectra of the Zn-OEP in DCM compared to the electropolymerized material deposited on the ITO at −1.0 V to 1.7 V, was in agreement with the behavior observed by Giraudeau et al. [13]. The Soret band experienced a redshift of 20 nm for the polymer, and the half-line width was broadened (Figure 3D). The spectra of the ZnOEP electropolymerized at 0.0 V to 1.6 V and 0.0 V to 2.2 V was similar to −1.0 V to 1.7 V, however, the Soret band half-line width was broader. In contrast, the absorption spectra of the ZnOEP deposited on the ITO with no electropolymerization displayed a splitting of the Soret band at 394 and 415 nm, indicating the presence of two different types of excitonic interactions between porphyrins. The UV-Vis absorption spectra of the NiOEP polymers showed a different picture. The spectra of the NiOEP electropolymerized at −1.0 to 1.7 V, resembling the UV-Vis spectrum of the NiOEP deposited on the ITO (no electropolymerization) and indicating that at this potential no polymer was being generated. However, the spectra of the NiOEP electropolymerized at 0.0 to 1.6 V and 0.0 to 2.2 V (Figure 3C) and resembled each other, displaying a splitting of the Soret band at 393 and 445 nm, again hinting at two different types of excitonic interactions. At the same time, the Q band of the NiOEP at 525 nm disappeared, and two new Q bands appeared around 555 and 615 nm. This result was in agreement with what was observed during the electropolymerization, where the polymer obtained between −1.0 and 1.7 V did not have a distinct coloration, but those obtained at the other two potential ranges showed a dark coloration. Finally, the UV-Vis spectra of the CuOEP polymerized under different conditions showed a half-line width broadening on all the ITO deposited materials compared to the DCM solution spectra, consistent with exciton coupling theory. The spectra of the ITO deposited the CuOEP and electropolymerized the CuOEP at −1.0 to 1.7 V and 0.0 to 1.6 V; they were very similar in displaying the Soret band at 394 nm with a half-line width of 30 nm. 

Interestingly, the Soret band for the electropolymerization at the 0.0 to 2.2 V range was red-shifted to 403 nm with a half-width of 40 nm, indicating that the higher potential is required in order to achieve electropolymerization with the CuOEP. 

The main difference in the spectra of the CuOEP polymers and monomers deposited on the ITO appeared in the B band between 520 and 540 nm (Figure 3A). The intensity of this band decreased in the CuOEP polymer obtained at the potential range of 0 V–1.6 V and was not observed when the polymer was obtained with the 0 to 2.2 V range. This behavior was observed for all metalloporphyrins polymers obtained at this potential (0 V to 2.2 V); in contrast, no significant changes occurred on the OEP polymers. (Figure 3A).

**Figure 4:**
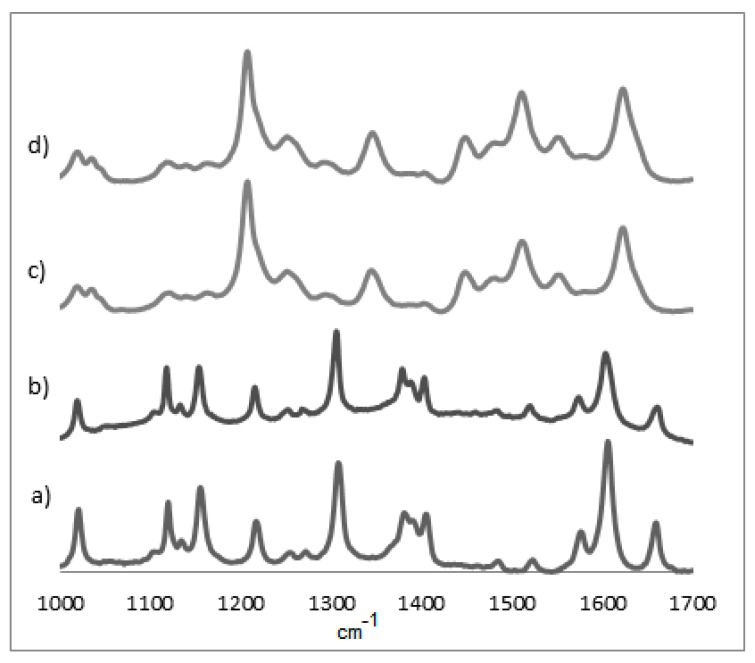
Raman spectra of NiOEP films on ITO surface: (**a**) reference porphyrin without electropolymerization; (**b**) potential range −1.0 V to 1.7 V; (**c**) potential range 0.0 V to 1.6 V; and (**d**) potential range 0.0 V to 2.2 V (excited at 532.0 nm).

The Raman spectra were obtained from 200 to 4000 cm^−1.^ However, frequencies from 1000 to 1700 cm^−1^ corresponding to porphyrin π-bond stretching vibrations and differences in this region are important to understand their electrochemical behavior. The NiOEP spectra are shown in Figure 4 and the other porphyrins can be found in the Supporting Information section, Figure S1. 

Using the Raman spectrum, the difference between the electropolymerized material and the individual monomers in the region between 1400 and 1500 cm^−1^ can be appreciated, as the absence of bands in this region hints at a successful polymerization. As seen in Figure 2, nickel does present some bands in this area (closer to 1500 cm^−1^) at all potentials, which would indicate a less extensive polymerization process. This could be also seen, but less clearly, in the UV-Vis at 400 nm for all cases for the NiOEP. This might explain the porphyrin’s fragile nature when it was later subjected to analysis with phenobarbital.

The principal Raman lines are shown in Table 3. The symmetric mode ν_2_ is mainly localized in the C_β_–C_β_ bond. The modes ν_10_ and ν_21_ are primarily C_α_–C_m_ stretching, ν_3_ results from almost equal contributions of C_β_–C_β_ and C_α_–C_m_ stretching and ν_4_ from C_α_–N stretching [23].

### 3.3. Analytical Performance

It was not possible to evaluate the performance of the electropolymerized NiOEP electrodes. The film formed was very thick and upon contact with the supporting electrolyte, some parts of the film detached from the surface of the ITO, indicating that such electrodes do not have mechanical stability and therefore are not useful for aqueous analysis. Most likely, the electropolymerization process gave a material with a different substitution pattern or a large difference in average molecular weight than for the other two metalloporphyrins.

**Figure 5:**
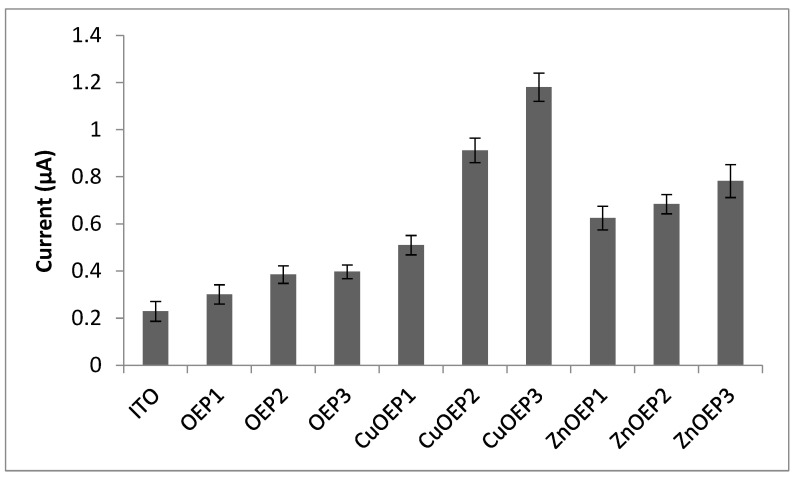
Analytical signal (n = 3) obtained on phenobarbital 0.001 mol · L^−1^ in LiClO_4_ 0.05 mol · L^−1^ by cyclic voltammetry with different modified electrodes. Potential range: 0.0 V–1.2 V (vs. Ag/AgCl), scan rate 0.1 V s^−1^. Electropolymerization conditions: (1) −1.0 to 1.7 V; (2) 0.0 to 1.6 V; and (3) 0.0 to 2.2 V.

The phenobarbital electroanalytical signals obtained for the OEP, CuOEP and ZnOEP polymers are compared in Figure 5.

Compared to the ITO electrode, all modified electrodes showed a better analytical response to phenobarbital. The electropolymerization of metalloporphyrins provides densely packed layers that facilitate the electron hopping process [10] and the metal contributes by intensifying the electron transfer. The CuOEP-modified electrodes obtained from 0.0 V to 1.6 V and 0.0 V to 2.2 V presented the highest analytical signals and the latter was used to demonstrate the applicability of the modified electrode to phenobarbital determination. The figures of merit for the analytical performance are shown in Table 4.

The calibration curve of the phenobarbital for the CuOEP^3^ electrode was obtained using the peak current at 0.564 V, as presented in the materials section. The phenobarbital concentration and current were linearly correlated between the LOD and 150 mg L^−1^ (R^2^ = 0.9957). The limits of detection (LOD) and quantification (LOQ) for the phenobarbital were determined according to the IUPAC (International Union of Pure and Applied Chemistry) directives [25].

For the determination of these values, the measurement of ten analytical blanks was carried out and the standard deviation of the measurements (σ) was calculated. The LOD and LOQ values were determined using the equations LD = *3σ/m* and LOQ = *10σ/m*, where *m* is the slope of the analytical curve. Considering the LOQ obtained, the method could be used in cases of intoxication where the phenobarbital concentrations found in blood are high. Adjustments to the sample volume can also be made to improve the LOD and LOQ.

Accuracy and precision, in terms of repeatability, were evaluated for the phenobarbital concentrations of 30.0, 60.0 and 118.0 mg L^−1^. The accuracy and precision (n = 3) range from 91.93% to 118.10% and from 3.48% to 5.07%, respectively. These accuracy and precision values are considered suitable for application on biological samples where precision may vary up to 15% along the analytical curve, except for points close to the LOQ where variation up to 20% is accepted and recoveries from 80% to 120% for the accuracy of the study [26].

The suggested method and electrode modifications would be suitable for the analysis of phenobarbital in human samples, as the ranges vary from 10 to 40 mg · L^−1^, typically found in antiepileptic treatments [27], to those at toxic (172–258 µM) or lethal levels (345– 650 µM) [28]. In general, the method described presents a much faster analysis time, a higher precision and similar accuracy to the chromatographic methods found in the literature. However, chromatographic methods generally present better LoDs [29,30].

## 4. Conclusions

The electropolymerization of octaethylporphyrin (OEP) and its metallo-complexes (Ni, Cu and Zn) with 4,4′-bypiridine was performed using cyclic voltammetry at three different potential ranges. The obtained polymers were characterized by UV-Vis spectroscopy and Raman spectroscopy. The minimum response current was obtained in the potential range from 0.0 to 2.2 V, indicating a better coverage of the ITO electrode surface at that potential range. The Raman bands were used to assess the level of electropolymerization and characteristic bands for the different polymerized porphyrins presented.

The potential for an analytical use of the modified electrodes to determine phenobarbital in aqueous solution has been evaluated for the first time. Our studies indicate that the electropolymerized CuOEP at potentials ranging from 0.0 to 2.2 V was the best performer. Given the good lineal range, precision and recovery values, the modified ITO electrode would be suitable for the analysis of phenobarbital in human samples.

## Figures and Tables

**Table 1:** Half-wave redox potentials (V vs. Ag/AgCl) of OEP and its metallo-complexes in different potential ranges of elecropolymerization (determined in the first cycle).

	Oxidation (V)		Reduction (V)	
Polymer	Dication	Radical Cation	Radical Anion	
OEP ^1^	1.56	1.15		−0.397; −0.813
OEP ^2^	1.59	1.05		
OEP ^3^	1.70	1.05		
CuOEP ^1^		1.00	0.535	−0.349; −0.781
CuOEP ^2^		0.972	0.486	0.137
CuOEP ^3^	1.968	0.972		
NiOEP ^1^	1.367	1.096	0.586	−0.427; −0.872
NiOEP ^2^	1.353	0.947	0.559	
NiOEP ^3^	1.343	0.945		
ZnOEP ^1^	1.235	0.732		0.061; −0.388; −0.737
ZnOEP ^2^	1.189	0.725		
ZnOEP ^3^	1.198 * 1.775	0.720		

1—Potential range −1.0 V to 1.7 V. 2—Potential range 0 to 1.6 V. 3—Potential range 0 to 2.2 V. *—Third oxidation peak.

**Table 2:** Spectroscopic data of the monomers and polymers obtained in different potential range.

	λ_max_ (nm)	
Modified Electrode	Soret Band	Q Bands
OEP (DCM)	399	498; 533; 567; 620
OEP *	381; 404	512.5 545; 574; 632
OEP ^1^	380; 410	513; 545; 574; 631
OEP ^2^	403	509; 546; 575; 630
OEP ^3^	399	507; 545; 574; 630
CuOEP (DCM)	398	525; 561
CuOEP *	393	541; 584
CuOEP ^1^	400	541, 584
CuOEP ^2^	394	536; 582
CuOEP ^3^	403	584
NiOEP (DCM)	392	516; 552
NiOEP *	382.5	525; 568
NiOEP ^1^	391	526; 568
NiOEP ^2^	393; 434	557; 616
NiOEP ^3^	393; 445	554, 614
ZnOEP (DCM)	401	531; 568
ZnOEP *	394; 415	548; 586
ZnOEP ^1^	416	543; 579
ZnOEP ^2^	418	546; 583
ZnOEP ^3^	416	582

* Monomer solution deposited on ITO, without electropolymerization. ^1^—Potential range −1 V to 1.7 V. ^2^—Potential range 0 to 1.6 V. ^3^—Potential range 0 to 2.2 V.

**Table 3:** Frequencies (cm^−1^) of selected Raman bands for the polymers/monomers of metallo-octaethylporphyrins on ITO (excited at 532.0 nm) at the different potential ranges.

Film	ν_21_	ν_4_	ν_3_	ν_2_	ν_10_
OEP *		1354	-	-	
OEP ^1^		1361	1541	1582	
OEP ^2^		1362	1539	1581	
OEP ^3^		1360	1540	1579	
CuOEP *	1310	1373	1499	1568 1580	1637
CuOEP ^1^	1312	1375	1502	1569 1584	1640
CuOEP ^2^	1313	1376	1514	1569 1585	1640
CuOEP ^3^	1311	1376	1504	1565 1581	1634
NiOEP *		1380	1522	1575	
NiOEP ^1^		1378	1519	1573	
NiOEP ^2^		1344	1511	1548	
NiOEP ^3^		1345	1510	1551	
ZnOEP *		1353	-	-	
ZnOEP ^1^		1347	1513	1551	
ZnOEP ^2^		1346	1513	1551	
ZnOEP ^3^		1347	1512	1551	

* Monomer solution deposited on ITO, without electropolymerization. ^1^—Potential range −1 V to 1.7 V. ^2^—Potential range 0 to 1.6 V. ^3^—Potential range 0 to 2.2 V.

**Table 4:** Analytical performance of CuOEP^3^ electrode to phenobarbital (PB) determination in aqueous solution.

Parameter	Value	
Linear range	10–150 mg L^−1^ (43.07–646 µM)	
R^2^	0.9957	
LOD	10 mg L^−1^ (43.07 µM)	
LOQ	35 mg L^−1^ (150.73 µM)	
PB conc. (mg/L)	Accuracy (% Recovery)	Precision (% RSD)
30 (129.2 µM)	118.10	3.48
60 (258.4 µM)	93.81	4.41
118 (508 µM)	91.93	5.07

## Data Availability

The data presented in this study are available on request from the corresponding author.

## Supplementary Materials

The following supporting information can be downloaded at: https://www.mdpi.com/article/10.3390/ma15196598/s1, Figures S1–S3: Raman spectra of the films on ITO surface.
