# Supplementary material for: Electropolymerization of Metallo-Octaethylporphyrins: A Study to Explore Their Sensing Capabilities
Source: Materials (Basel). 2022 Sep 23;15(19):6598. doi: 10.3390/ma15196598 (PMC9571886; doi:10.3390/ma15196598)
Supplement: Supplementary file 1 [file materials-15-06598-s001.zip › materials-1926677-supplementary.pdf]

Supporting Information

Electropolymerization of Metallo-Octaethylporphyrins: A Study to Explore Their Sensing Capabilities

Clésia C. Nascentes [1], Ivette Aguilar [2], Guzmán Gil-Ramírez [2] and Jose Gonzalez-Rodriguez [2,*]

[1] Department of Chemistry, Federal University of Minas Gerais, P.O. Box 702, Belo Horizonte 31270-901, Brazil
[2] School of Chemistry, University of Lincoln, Brayford Pool, Lincoln LN6 7TS, UK
* Correspondence: jgonzalezrodriguez@lincoln.ac.uk; Tel.: +44-1522886878

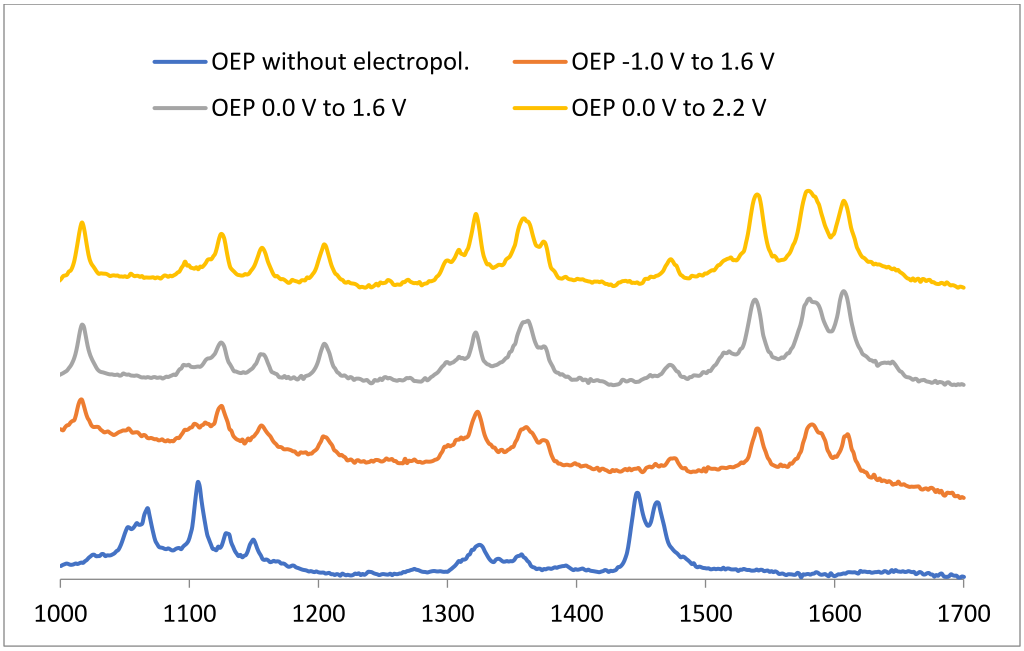

Figure S1. Raman spectra of OEP films on ITO surface: without electropolymerization; potential range -1.0 V to 1.7 V; potential range 0.0 V to 1.6 V and potential range 0.0 V to 2.2 V (excited at 532.0 nm).

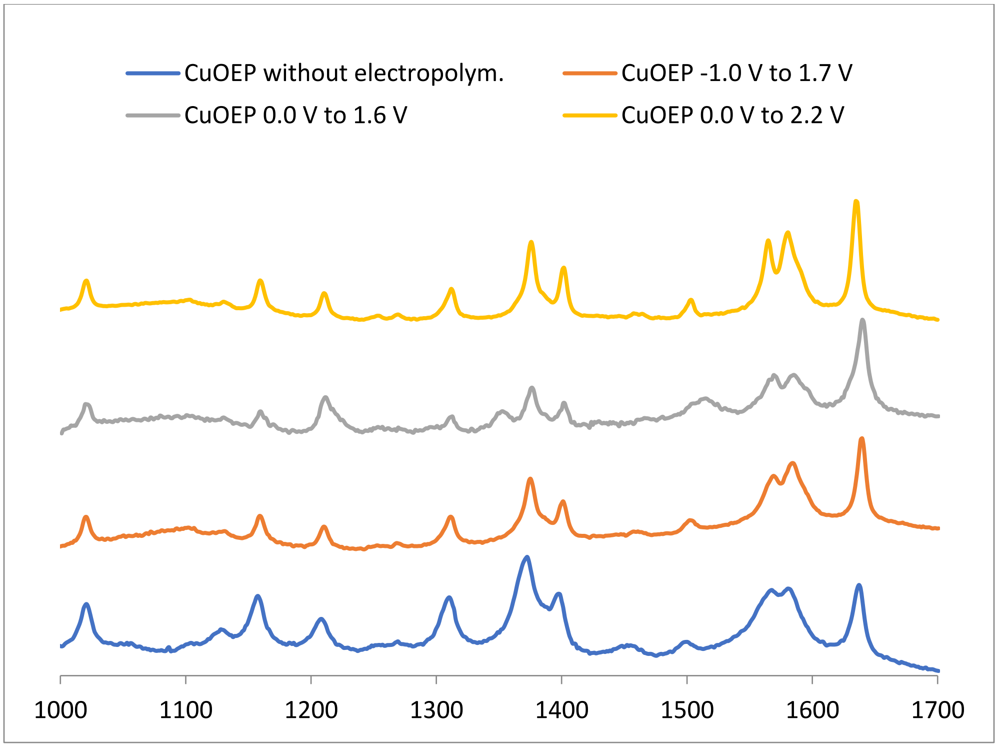

Figure S2. Raman spectra of CuOEP films on ITO surface: without electropolymerization; potential range -1.0 V to 1.7 V; potential range 0.0 V to 1.6 V and potential range 0.0 V to 2.2 V (excited at 532.0 nm).

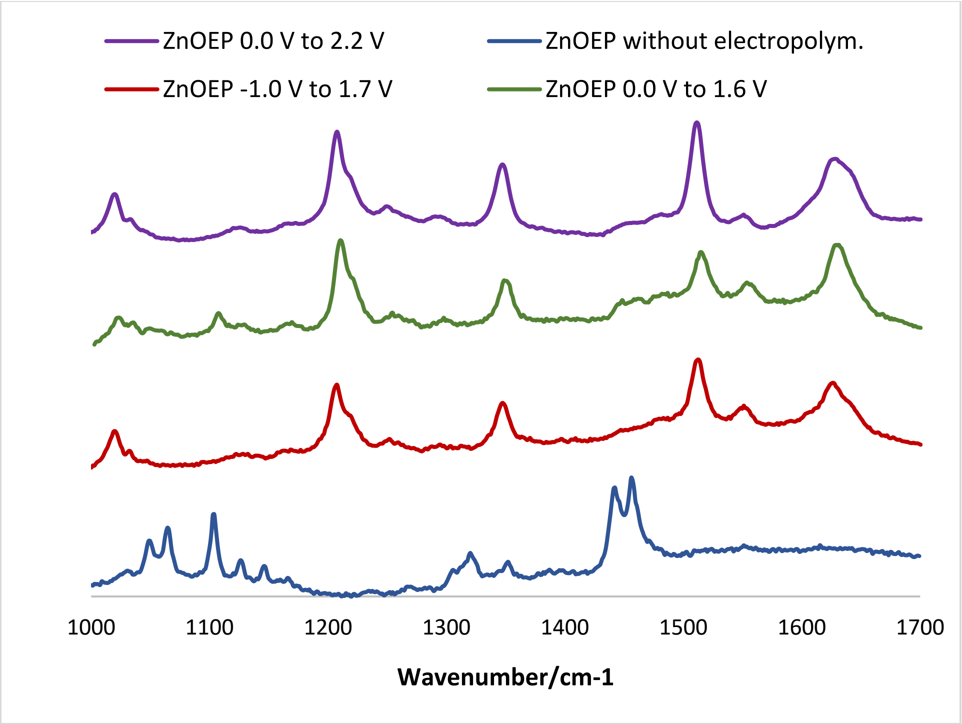

Figure S3. Raman spectra of ZnOEP films on ITO surface: without electropolymerization; potential range -1.0 V to 1.7 V; potential range 0.0 V to 1.6 V and potential range 0.0 V to 2.2 V (excited at 532.0 nm).
